# DISPARITIES IN ALZHEIMER’S DISEASE: THE ROLE OF REPEATED ANESTHETIC AND SURGICAL EXPOSURE

**DOI:** 10.1093/geroni/igz038.1594

**Published:** 2019-11-08

**Authors:** Miklos D Kertai

**Affiliations:** Vanderbilt University Medical Center, Nashville, Tennessee, United States

## Abstract

Annually, there are 7 million patients > 65 years who undergo noncardiac surgery in the US. This number is expected to increase by 30% over the next 3 decades, and given the prevalence of surgically correctable comorbidities, the elderly will continue to undergo multiple surgical procedures. Currently, it is unclear what factors initiate or promote the development of Alzheimer’s disease (AD). Preclinical studies suggest that exposure to anesthesia and/or surgery could be one of the environmental exposures that increase AD risk through neuroinflammation and neuroapoptosis. However, previous studies indicated substantial disparities in AD risk by gender, ethnicity, and race, but failed to explore the role of anesthesia and/or surgery exposure in the risk for AD. This presentation will review the role of disparities and anesthesia and/or surgery exposure in the risk for AD in elderly surgical patients.

